# Differential Impact of Pneumococcal Conjugate Vaccines on Hospitalized *Versus* Outpatient Community-Acquired Alveolar Pneumonia in Children Younger Than 5 Years Suggests Differences in Pathogenesis

**DOI:** 10.1093/ofid/ofaf710

**Published:** 2025-11-18

**Authors:** Bart A van der Beek, David Greenberg, Guy Hazan, Ron Dagan

**Affiliations:** The Shraga Segal Department of Microbiology, Immunology and Genetics, Faculty of Health Sciences, Ben Gurion University of the Negev, Beer Sheva, Israel; Pediatric Division, Faculty of Health Sciences, Ben Gurion University of the Negev, Beer Sheva, Israel; Pediatric Infectious Diseases Unit, Soroka University Medical Center, Beer Sheva, Israel; Pediatric Pulmonary Unit, Soroka University Medical Center, Beer Sheva, Israel; The Shraga Segal Department of Microbiology, Immunology and Genetics, Faculty of Health Sciences, Ben Gurion University of the Negev, Beer Sheva, Israel

**Keywords:** pneumococcal conjugate vaccines, pneumonia, pediatrics, hospitalization, outpatient

## Abstract

**Background:**

Current evidence suggests that in young children with community-acquired alveolar pneumonia (CAAP), bacterial-viral coinfections (mostly respiratory syncytial virus [RSV]-pneumococcus coinfections) are more prevalent among hospitalized children than among outpatients and that RSV-pneumococcal coinfections are more frequently associated with non-PCV13 serotypes. Based on this background, we speculated that following pneumococcal conjugate vaccine [PCV] implementation, the decline of hospitalized CAAP episodes would be lower than that of outpatient episodes.

**Methods:**

This analysis was a part of an ongoing (since 2004) population-based, active surveillance in children < 5 years, including all CAAP visits to the pediatric emergency room in southern Israel. Community-acquired alveolar pneumonia was radiologically confirmed by consensus reading. Episodes were divided into hospitalized and those discharged without hospitalization (outpatients). We used a negative binomial regression model to evaluate PCV7/PCV13 impact by age and ethnic group using monthly and yearly incidence rates. Analyzed periods were pre-PCV (2004–2009), PCV7/PCV13 transition (2009–2011), early-PCV13 (2011–2015), and late-PCV13 (2015–2019).

**Results:**

Of 11 130 episodes, 3677 and 7633 were outpatients and hospitalized, respectively. Post-PCV incidence dynamics significantly diverged between the 2 study groups. (1) During the PCV7/PCV13 transition, outpatient rates significantly declined, but not those of hospitalizations. 2) During late-PCV13 period, a significantly greater decline was reached among outpatients (67%; 95% confidence interval [CI] 62%–71%) versus hospitalized (47%; 95% CI 41%–54%). This difference remained for all age and ethnic groups.

**Conclusions:**

The marked divergence in PCV impact between hospitalized and outpatient episodes is intriguing, but consistent with previous studies suggesting that hospitalized CAAP was associated with a lower proportion of PCV13 serotypes, in conjunction with viral-bacterial (mostly RSV-pneumococcus) coinfections.

## Teaser

Following pneumococcal conjugate vaccine implementation, rates of hospitalized episodes of community-acquired alveolar pneumonia in young children were reduced to a lower extent than those of outpatients, reflecting the predicted lower proportion of vaccine serotype pneumococci and higher proportion of viral-bacterial coinfection.

Pneumonia is a major global cause of morbidity and mortality in children under 5 years of age, with approximately 156 million new cases and 20 million hospitalizations annually [1–4]. Establishing the etiology of pneumonia is challenging and often not feasible. Pneumonia episodes may often be caused by multiple pathogens and frequently involve viral-bacterial coinfections [5–9]. Radiologically confirmed community-acquired alveolar pneumonia (CAAP) is considered the most specific endpoint for evaluating pneumococcal etiology, especially in studies of the efficacy, effectiveness, and impact of pneumococcal conjugate vaccines (PCVs) [5, 10–13]. Among all radiological pneumonia endpoints, CAAP shows the highest inter-observer agreement and the greatest specificity for pneumococcal etiology [13–15].

The important role of *Streptococcus pneumoniae* in CAAP among young children has been demonstrated through the observed impact of PCV implementation on CAAP incidence, in so-called “probe studies” [16–20]. These studies highlight not only the contribution of *S. pneumoniae*, but also suggest that a large proportion of pneumococcal CAAP is caused by serotypes included in the PCVs (vaccine-type [VT] serotypes). However, vaccine probe studies and other observational studies have shown that, beyond *S. pneumoniae*, respiratory viruses—especially respiratory syncytial virus (RSV)—play an important role as coinfecting agents in CAAP.

Pneumococcal conjugate vaccines may reduce virus-positive CAAP, while reduced circulation of respiratory viruses (as observed during the COVID-19 pandemic) can also decrease pneumococcal disease, including CAAP [6, 7, 21–24]. Respiratory syncytial virus is the most common and impactful virus contributing to CAAP in young children [6, 7, 22]. Among children with CAAP, carriage of non-vaccine pneumococcal serotypes (non-VT) is more common in those coinfected with RSV than in those without detected viruses or with other respiratory viruses [25]. Additionally, young children hospitalized for CAAP more frequently showed clinical signs and laboratory findings suggesting viral coinfections than those seen at the pediatric emergency room (PER), but subsequently discharged (outpatients) [26]. Based on these findings, we hypothesized that following PCV implementation, hospitalization rates for CAAP would decline less than those for outpatient CAAP, as hospitalized cases are more likely to be coinfections with respiratory viruses and with a high rate of non-VT pneumococci.

In southern Israel (the Negev district), >95% of births occur in one hospital (Soroka University Medical Center [SUMC]) where essentially all PER visits and hospitalizations occur, allowing incidence calculation. As part of an ongoing population-based prospective study in southern Israel, we have been monitoring all PER visits and the resulting hospitalizations where chest radiographs (CXR) were obtained [5]. This ongoing population-based prospective surveillance initiated in 2004 (5 years before PCV implementation) has enabled us to study the impact of PCVs on CAAP incidence dynamics in southern Israel and compare trends between hospitalized and outpatient episode incidence.

## MATERIALS AND METHODS

This analysis is part of an ongoing prospective, population-based active surveillance. The analyzed data span from July 2004 through June 2019. The study was approved by the institutional ethics committee of SUMC.

### Setting

Soroka University Medical Center is the only hospital in the Negev district of southern Israel, where ∼95% of the region's children are born. It provides both primary and referral health services to the region's population (∼760 000 inhabitants and ∼95 000 children under 5 years old in 2019) [27] enabling population-based research and incidence calculations in young children.

Two distinct ethnic populations live side by side in the region: the Bedouin and the Jewish populations. The Bedouin population, transitioning from a semi-nomadic to an urban lifestyle, shares characteristics with developing populations, while the Jewish community is predominantly urban and resembles a developed population overall [28]. During the study period, the mean annual number of births at SUMC was similar between the 2 ethnic groups: 9373 Jewish and 10 430 Bedouin children in 2019 [29]. The Bedouin population had higher hospitalization rates for respiratory infectious illnesses, including CAAP [19, 30–32]. As medical insurance for children in Israel is universal and free of charge, there are no financial barriers to accessing healthcare services in the region.

### Study Design

All children visiting SUMC with respiratory infections are initially seen at the PER. After examination (including CXR if deemed necessary), a decision is made to either hospitalize or discharge the child. Chest radiography may also be performed after hospital admission. The study included (1) all children < 5 years of age residing in the Negev district who visited SUMC; (2) had a CXR within <48 h of arrival (either at the PER or after admission to the hospital); and (3) were diagnosed by the research team as radiologically confirmed CAAP according to the WHO Pneumonia Working Group definition [10, 12, 15]. Children with radiologically confirmed CAAP were further classified as either hospitalized or discharged from the PER without hospitalization (outpatient).

Each day, 2 pediatric infectious disease specialists evaluated every CXR, independently, unaware of the clinical data and the assessment of the clinical team. An independent pediatric radiologist conducted an additional evaluation. Radiologically confirmed CAAP was defined by agreement between at least one of the study's pediatric infectious disease specialists and the study pediatric radiologist. Variables recorded for this analysis included the date of diagnosis, age, ethnicity (Jewish or Bedouin), and hospitalization status (admitted or discharged). Two consecutive episodes were considered distinct if separated by at least 28 days from the diagnosis of the first.

### Vaccine Uptake

Estimates of PCV7 coverage before 2009 were based on sales figures provided by the distributor. In 2007–2008, ∼25% of Jewish and <5% of Bedouin children aged 12–23 months had received at least 2 PCV doses. The methodology of evaluating vaccine uptake has been described elsewhere [33]. Since 2013, approximately 95% and 90% of all children under 3 years of age had received 2 and 3 doses, respectively, with similar rates among children of the 2 ethnic populations.

### Statistical Analysis

A negative binomial regression model was used to evaluate the impact of PCV implementation on CAAP by age and ethnic group. Separate models were fitted for each time series. An offset variable representing the population at risk per month was introduced to standardize the analysis by adjusting for changes in population over time.

To capture seasonal patterns inherent in the data, trigonometric functions (sines and cosines) were added to the model. Post-vaccine changes were modeled using a series of indicator variables representing step changes in each post-PCV period. The counterfactual, representing the expected number of cases in the absence of PCV introduction, was calculated from the fitted model while setting the parameters for the post-PCV step changes to 0.

We divided the study period into four distinct periods: pre-PCV (July 2004–June 2009), PCV7/PCV13 transition (July 2009–June 2011), early-PCV13 (July 2011–June 2015), and late-PCV13 (July 2015–June 2019). The late-PCV13 period was selected based on prior evidence indicating that, after an initial decline, rates of post-PCV invasive pneumococcal disease (IPD) and respiratory illnesses stabilized around 2015 [5, 22, 34]. Rate ratios and their corresponding 95% confidence intervals (95% CI) were calculated by dividing the predicted values (ie, modeled estimates based on observed data) for each vaccine period by the counterfactual values estimated under the assumption of no vaccine introduction.

All analyses were conducted in R version 4.3.3.

## RESULTS

**Figure 1.**
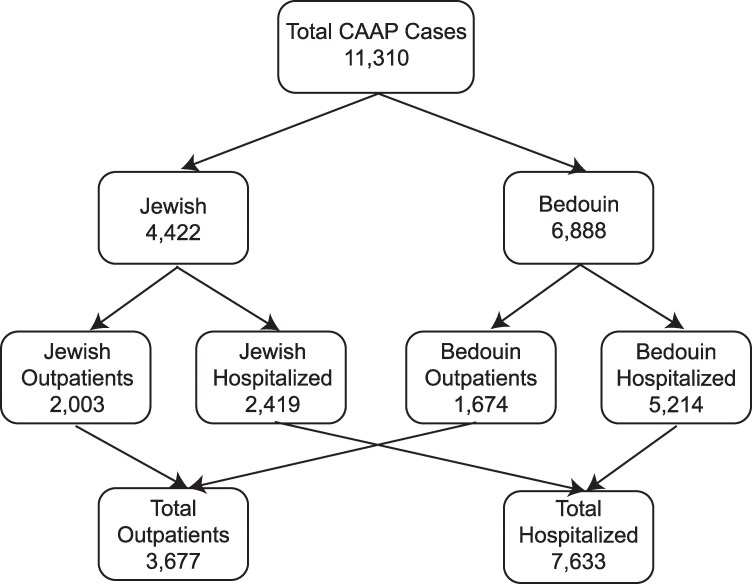
CAAP cases in children under 60 months per ethnicity and case setting: July 2004–June 2019.

A total of 9891 children < 60 months with 11 310 episodes of CAAP were reported during the study period (July 2004 through June 2019) (Figure 1). More than one episode occurred in 1038 (10.5%) children: 2 episodes in 898 (8.1%) children (mean interval between episodes 421.9 days), 3 episodes in 256 (1.6%) children (mean interval 328.4 days), and ≥4 episodes in 84 (0.8%) children (mean interval 279.0 days). Of these, 6888 episodes (60.9%) occurred among Bedouin children and 4422 episodes (39.1%) among Jewish children. A total of 7633 (67.5%) episodes required hospitalization: 5214 (75.7%) among Bedouin children and 2419 (54.7%) among Jewish children. The distribution of episodes across the 3 age groups (<12 months, 12–23 months, and 24–59 months) is presented in Supplementary Figure 1.

During the pre-PCV period, the mean incidence (per 1000 child-years) of hospital visits for CAAP was 13.3 ± 0.7. The mean incidence among Bedouin children was 1.6 times higher than that among Jewish children (16.4 ± 1.0 vs 10.3 ± 1.6, respectively) (Table 1). The largest difference between these 2 groups was observed in children < 12 months (mean incidence of 36.2 ± 5.1 and 12.6 ± 2.3, respectively), while in other age groups, the difference was smaller, though still significantly higher among Bedouin children.

**Table 1.** Mean Annual CAAP Incidence per 1000 Children < 60 Months Across Vaccine Periods (July 2004–June 2019, by Ethnicity and Care Setting)

	Outpatients			Hospitalized			Total		
	Jewish	Bedouin	Total	Jewish	Bedouin	Total	Jewish	Bedouin	Total
2004–2005	5.5	4.8	5.2	3.5	10.7	7.0	9.0	15.5	12.2
2005–2006	5.5	4.4	5.0	4.9	11.8	8.3	10.5	16.2	13.3
2006–2007	5.3	5.4	5.3	4.6	12.2	8.3	9.9	17.6	13.6
2007–2008	7.1	4.7	5.9	5.9	10.8	8.2	13.0	15.5	14.2
2008–2009	4.5	4.5	4.5	4.8	12.9	8.7	9.3	17.4	13.2
Mean 2004–2009	5.6 ± 0.9	4.8 ± 0.4	5.2 ± 0.5	4.7 ± 0.9	11.7 ± 0.9	8.1 ± 0.6	10.3 ± 1.6	16.4 ± 1.0	13.3 ± 0.7
2009–2010	3.8	4.0	3.9	6.3	13.3	9.7	10.1	17.4	13.6
2010–2011	3.6	2.9	3.3	5.2	11.9	8.4	8.7	14.8	11.7
Mean 2009–2011	3.7 ± 0.2	3.5 ± 0.8	3.6 ± 0.5	5.7 ± 0.8	12.6 ± 1.0	9.0 ± 0.9	9.4 ± 1	16.1 ± 1.8	12.6 ± 1.4
2011–2012	2.7	1.9	2.3	3.6	7.5	5.5	6.3	9.4	7.8
2012–2013	1.9	1.6	1.8	2.8	7.4	5.0	4.6	9.1	6.8
2013–2014	1.7	1.8	1.7	3.2	6.2	4.7	4.9	7.9	6.4
2014–2015	1.8	1.5	1.6	2.8	6.4	4.6	4.6	7.9	6.2
Mean 2011–2015	2.0 ± 0.5	1.7 ± 0.2	1.8 ± 0.3	3.1 ± 0.4	6.9 ± 0.7	4.9 ± 0.4	5.1 ± 0.8	8.5 ± 0.8	6.8 ± 0.7
2015–2016	2.3	2.0	2.1	2.7	7.4	5.0	4.9	9.4	7.1
2016–2017	1.5	2.0	1.8	3.5	7.0	5.2	5.0	9.0	7.0
2017–2018	1.4	1.5	1.5	2.6	5.6	4.1	4.0	7.1	5.5
2018–2019	2.0	1.4	1.7	3.6	5.5	4.5	5.6	6.9	6.2
Mean 2015–2019	1.8 ± 0.4	1.7 ± 0.3	1.8 ± 0.3	3.1 ± 0.5	6.3 ± 1.0	4.7 ± 0.5	4.9 ± 0.6	8.0 ± 1.3	6.4 ± 0.7

The mean incidence of episodes was generally higher in hospitalized children than in those who were discharged (outpatients) (8.1 ± 0.6 vs 5.2 ± 0.5, respectively). While the rate of outpatient visits was of the same order of magnitude among Bedouin and Jewish children, the incidence of hospitalized episodes was 2.5 times higher among Bedouin children (11.7 ± 0.9 vs 4.7 ± 0.9). This difference was the highest in children< 12 months (6.1 ± 1.6 vs 4.4 ± 1.2, and 30.2 ± 3.6 vs *8*.2 ± 1.4, respectively, for outpatients and hospitalizations) (Table 1; Supplementary Table 1).

During the PCV7/PCV13 transition period, overall CAAP rates did not change significantly (Table 1; Supplementary Table 1). However, the trends in hospitalized versus outpatient episode rates significantly diverged: while the outpatient episode incidence rates significantly declined, those of hospitalized episodes increased, but not significantly. Following PCV13 implementation, a rapid reduction in both outpatient and hospitalized episode rates was observed during the early-PCV13 period, followed by stabilization during the late-PCV13 period. Trends during all post-PCV implementation periods were similar across all age groups and both ethnic populations.

**Figure 2.**
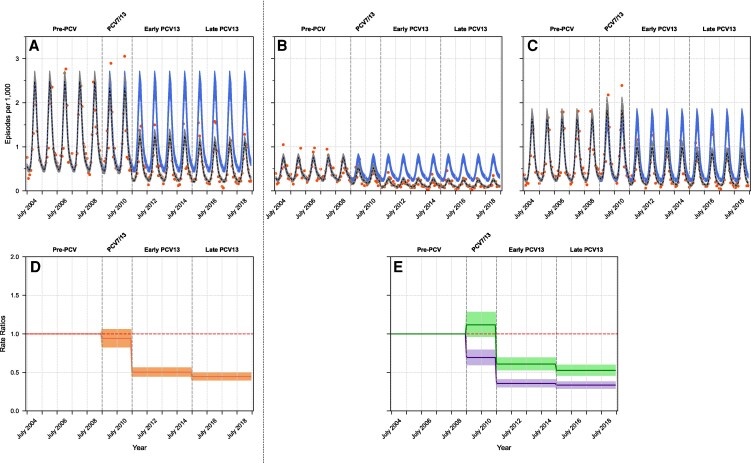
Monthly CAAP incidence in children < 60 months of age. *A–C*, Comparison of observed versus expected (counterfactual) incidence trends during the post-PCV implementation periods, transition, early-PCV13, and late-PCV13, across care settings: (*A*) overall, (*B*) outpatient, and (*C*) hospitalized. Orange dots represent the observed incidence. The black dashed line shows the model-predicted incidence, with the gray-shaded area indicating the 95% CI. The blue solid line represents the expected incidence with a light blue-shaded area showing the 95% CI. *D* and *E*, IRRs for each post-PCV period compared to the pre-PCV period across care settings: (*D*) overall and (*E*) outpatients and hospitalized. The colored line shows the IRRs, with a shaded area representing the 95% CI (orange, overall; purple, outpatients; green, hospitalized). A red dashed horizontal line at IRR = 1.0 marks the threshold between reductions and increases in incidence.

To determine the extent of incidence decline, we compared the modeled incidence rates based on the observed results with the modeled expected values based on the pre-PCV period (Table 2 and Figure 2). This was expressed as incidence rate ratios (IRRs) and 95% CIs comparing observed and expected mean incidence rates for each post-implementation period. In the PCV7/PCV13 transition period, no significant decline was observed in overall CAAP visit rates (IRR 0.94 [0.83; 1.03]). However, divergent trends were seen when outpatient and hospitalized episodes were analyzed separately: outpatient episodes declined significantly (IRR 0.69 [0.60; 0.79]), while hospitalized episodes showed a non-significant increase (IRR 1.12 [0.97; 1.28]). This difference between outpatient and hospitalized episodes was statistically significant. During the early-PCV13 and late-PCV13 periods, all episodes significantly declined: IRR 0.50 (0.45; 0.56) and 0.45 (0.40; 0.49), respectively. The IRRs were significantly lower for outpatient than for hospitalized episodes: 0.36 (0.31; 0.41) versus 0.61 (0.53; 0.69), respectively, during the early-PCV period; and 0.33 (0.29; 0.37) versus 0.53 (0.46; 0.59), respectively, during the late-PCV13 period.

**Table 2.** Mean IRRs With 95% Confidence Intervals for Every Vaccine Period vs Expected Episodes in Children < 60 Months, by Care Setting, Ethnic Group, and Age Group

	7/13 Transition Period	Early PCV13 Period	Late PCV 13 Period
<12 m			
Outpatients			
Jewish	0.57 (0.39; 0.81)	0.39 (0.28; 0.53)	0.42 (0.30; 0.58)
Bedouin	0.68 (0.48; 0.94)	0.28 (0.20; 0.38)	0.41 (0.30; 0.55)
Overall	0.63 (0.48; 0.81)	0.32 (0.25; 0.42)	0.41 (0.33; 0.52)
Hospitalized			
Jewish	1.06 (0.79; 1.39)	0.71 (0.55; 0.91)	0.83 (0.66; 1.08)
Bedouin	1.06 (0.84; 1.31)	0.59 (0.48; 0.72)	0.53 (0.43; 0.65)
Overall	1.05 (0.86; 1.26)	0.62 (0.52; 0.73)	0.60 (0.51; 0.72)
All episodes			
Jewish	0.87 (0.68; 1.09)	0.58 (0.47; 0.71)	0.68 (0.55; 0.84)
Bedouin	0.98 (0.79; 1.20)	0.53 (0.44; 0.63)	0.50 (0.41; 0.60)
Overall	0.94 (0.79; 1.12)	0.54 (0.47; 0.63)	0.56 (0.47; 0.65)
12–23 m			
Outpatients			
Jewish	0.54 (0.41; 0.69)	0.33 (0.26; 0.41)	0.35 (0.27; 0.44)
Bedouin	0.57 (0.44; 0.73)	0.31 (0.24; 0.40)	0.29 (0.23; 0.37)
Overall	0.56 (0.46; 0.66)	0.32 (0.27; 0.38)	0.32 (0.27; 0.38)
Hospitalized			
Jewish	1.19 (0.91; 1.50)	0.60 (0.47; 0.75)	0.61 (0.48; 0.76)
Bedouin	0.94 (0.76; 1.14)	0.48 (0.40; 0.58)	0.46 (0.38; 0.56)
Overall	1.02 (0.86; 1.20)	0.53 (0.45; 0.61)	0.51 (0.43; 0.60)
All episodes			
Jewish	0.78 (0.64; 0.94)	0.43 (0.36; 0.51)	0.45 (0.37; 0.53)
Bedouin	0.80 (0.67; 0.94)	0.42 (0.36; 0.49)	0.40 (0.33; 0.47)
Overall	0.79 (0.69; 0.90)	0.43 (0.38; 0.48)	0.42 (0.37; 0.47)
24–59 m			
Outpatients			
Jewish	0.79 (0.62; 0.98)	0.37 (0.29; 0.46)	0.26 (0.21; 0.33)
Bedouin	0.87 (0.69; 1.11)	0.43 (0.34; 0.55)	0.36 (0.28; 0.45)
Overall	0.83 (0.69; 0.98)	0.40 (0.33; 0.46)	0.30 (0.25; 0.36)
Hospitalized			
Jewish	1.20 (0.94; 1.51)	0.58 (0.46; 0.73)	0.42 (0.33; 0.53)
Bedouin	1.26 (1.02; 1.54)	0.65 (0.54; 0.79)	0.42 (0.35; 0.51)
Overall	1.23 (1.04; 1.45)	0.63 (0.54; 0.73)	0.42 (0.35; 0.50)
All episodes			
Jewish	0.86 (0.73; 1.00)	0.48 (0.41; 0.54)	0.44 (0.38; 0.51)
Bedouin	1.00 (0.86; 1.16)	0.52 (0.45; 0.60)	0.45 (0.39; 0.51)
Overall	0.94 (0.83; 1.05)	0.50 (0.45; 0.56)	0.45 (0.40; 0.49)
All children			
Outpatients			
Jewish	0.66 (0.55; 0.78)	0.36 (0.30; 0.42)	0.32 (0.27; 0.38)
Bedouin	0.73 (0.61; 0.87)	0.36 (0.30; 0.42)	0.35 (0.30; 0.41)
Overall	0.69 (0.60; 0.79)	0.36 (0.31; 0.41)	0.33 (0.29; 0.38)
Hospitalized			
Jewish	1.13 (0.93; 1.35)	0.64 (0.53; 0.75)	0.60 (0.50; 0.71)
Bedouin	1.13 (0.95; 1.33)	0.59 (0.51; 0.69)	0.49 (0.42; 0.57)
Overall	1.12 (0.97; 1.28)	0.61 (0.53; 0.69)	0.53 (0.46; 0.59)
All episodes			
Jewish	0.69 (0.60; 0.79)	0.36 (0.31; 0.41)	0.33 (0.29; 0.38)
Bedouin	1.12 (0.97; 1.28)	0.61 (0.53; 0.69)	0.53 (0.46; 0.59)
Overall	0.94 (0.83; 1.06)	0.50 (0.45; 0.56)	0.45 (0.40; 0.49)

The post-PCV implementation trends across the 3 periods were similar across all age groups and both ethnic groups (Supplementary Figures 2–9 and Tables 1 and 2). Notably, mean IRRs did not differ significantly between Jewish and Bedouin children, despite the considerable and significant difference in CAAP incidence rates between the 2 groups during the pre-PCV period. Additionally, in older age groups, the mean IRRs during the late-PCV13 period tended to be slightly lower than during the early-PCV13 period, although the difference was not statistically significant.

## DISCUSSION

The results of the current study show clearly that following PCV implementation, hospital visits for CAAP in young children in southern Israel declined rapidly and significantly. However, the rate dynamics differed significantly between hospitalized children and those discharged from the PER (outpatients). First, during the PCV7/PCV13 transition period, outpatient episode rates declined, while those of hospitalizations increased, although not significantly. Second, during the early- and late-PCV13 periods, while both groups showed a deep and significant decline compared to the pre-PCV period, the decline in outpatient episodes was significantly steeper. At first glance, these findings may seem counterintuitive since vaccines are expected to protect better against severe disease than against mild disease (assuming that hospitalized episodes in general are more severe). However, some of our previously published studies provide a plausible explanation for these findings.

An early, pre-PCV implementation study suggested that the serotypes most likely to progress to CAAP, when carried in healthy children, were serotypes 1, 5, 7F, 9V, 14, 19A, and 22F [35]. All except serotype 22F are included in PCV13 and serotypes 1, 5, 7F, and 19A are not included in PCV7. This study was followed by a study assessing the early impact of PCV7 and PCV13 implementation on pneumococcal bacteremic pneumonia versus non-pneumonia IPD [36]. PCV7 was introduced in July 2009 (with catch-up in children < 24 months) and replaced by PCV13 16 months later. During the PCV7/PCV13 transition period, most of the observed impact was attributable to PCV7. During this period, episodes of pneumococcal bacteremic pneumonia did not decline (IRR 0.94; 95% CI, .73–1.30), whereas episodes of non-pneumonia IPD declined sharply (IRR.0.51, 95% CI, .41–.63). Further analysis showed that PCV7 serotypes declined significantly and to a similar extent in both groups, but a sharp increase of serotypes 1, 5, and 19A (not in PCV7) among bacteremic pneumonia cases accounted for the difference between the groups. Shortly after the replacement of PCV7 by PCV13, both pneumococcal bacteremic pneumonia and non-pneumonia IPD declined.

A subsequent study assessed the early impact of PCV7/PCV13 implementation on non-bacteremic CAAP hospital visits [19]. During the short PCV7/PCV13 transition period, rates of hospitalized CAAP increased non-significantly, while outpatients’ rates significantly decreased by 42%. A follow-up study showed that the 3 serotypes, 1, 5, and 19A, caused more severe CAAP than other serotypes [37]. The predilection of serotypes 1, 5, 7F, and 19A to cause pneumonia, along with the greater severity of disease associated with these serotypes compared to other serotypes in PCV7/PCV13, could provide a plausible explanation, at least in part, to the different dynamics in hospitalized versus outpatient episodes during the post-PCV7/PCV13 transition period and aligns with the declining trend in both hospitalized and outpatient episodes. However, although both outpatient and hospitalized episodes declined, the reduction in outpatient episodes was significantly greater during both the early and late post-PCV13 periods. These differences persisted across both ethnic groups and all age categories.

Can the differences in dynamics between the outpatient and hospitalized episodes be explained? Once again, previous studies from our region may offer a plausible explanation. First, we compared the clinical characteristics of hospitalized children and outpatients during the pre-PCV period (2004–2009). This study was a nested prospective study, done on the pre-PCV cohort within our CAAP surveillance described in the current publication, comparing demographic and clinical characteristics between hospitalized and outpatient children. All had radiologically confirmed CAAP, but hospitalized cases were younger and had significantly lower mean temperatures, lower peripheral white blood cell and neutrophil counts, and lower C-reactive protein (CRP) levels. In contrast, they had significantly higher rates of hypoxemia, cough, and respiratory virus detection. Although the 2 groups differed significantly in the described characteristics, both had radiologically confirmed CAAP and had abnormally high mean CRP, white blood cell count and neutrophil count values, and mean temperature on admission, suggesting that most had bacterial infections. Thus, the findings suggested that the hospitalized children could have more frequently bacterial-viral coinfections whereas outpatients exhibited more “classical” bacterial features [26]. Furthermore, CAAP episodes occurring during the viral respiratory season were associated with a 1.83 times higher risk of hospitalization compared to those occurring outside the respiratory season [26]. Although the findings discussed above strongly suggest more frequent viral-pneumococcal coinfections among hospitalized patients with CAAP, at least some hospitalizations due to pure viral CAAP cannot be ruled out, contributing to a lower PCV impact, especially in young infants.

Second, an additional study on a subset of hospitalized children from the current surveillance showed that CAAP episodes in children < 5 years associated with serotypes 1, 5, 7F, 14, and 19A (included in PCV13) had higher odds of fever ≥ 39°C, peripheral blood cell counts ≥ 20 000/µL, and serum CRP concentrations ≥ 70 µg/L, but lower odds of hypoxemia and respiratory virus detection [38]. These findings suggest that the clinical characteristics of the outpatients described above [26] derive, at least in part, from a high proportion of these PCV13 serotypes, while they are likely to constitute a lesser proportion in hospitalized cases.

Third, epidemiological studies covering the periods before and during the COVID-19 pandemic showed that both respiratory viruses and pneumococci contributed to CAAP cases in children < 60 months, often as coinfections, with RSV being by far the dominant respiratory virus involved [6, 7, 38, 39]. On the other hand, PCVs significantly impacted RSV-positive pneumonia as demonstrated in both efficacy studies and post-implementation [22–24]. In a study conducted on a subgroup of hospitalized children from the current surveillance, PCV13 implementation had a substantial impact on both all-CAAP and RSV-positive CAAP hospitalizations of children < 60 months, demonstrating the importance of RSV-pneumococcal coinfections in CAAP among young children [22]. The demonstration of frequent mutual synergetic effects of RSV and pneumococcus in CAAP strongly suggests that RSV-pneumococcus coinfections constitute an important component of the hospitalized young children with CAAP in our region, although additional pure viral cases cannot be ruled out.

Fourth, a nested study within our surveillance, examining hospitalized CAAP episodes in children < 60 months during the pre-PCV period, showed that children with CAAP with concomitant nasopharyngeal carriage of PCV13 serotypes (serotypes 1, 4, 5, 6A, 6B, 7F, 14, 9 V, and 19A grouped) were significantly less likely to be coinfected with RSV compared to those carrying non-PCV13 serotypes such as 33F, 17F, 15B, 15C, and 35B [25]. Support to these findings comes from a recently presented French study, where IPD cases during the RSV season were more likely to be caused by noninvasive serotypes, in contrast to cases during the non-RSV season, which were more frequently caused by invasive serotypes [40]. In that study, most of the noninvasive serotypes were non-PCV13, whereas most of the invasive serotypes were PCV13 serotypes.

In conclusion, a marked divergence in PCV impact between hospitalized and outpatient-managed children < 5 years old was observed, consistent with multiple previous observations suggesting differences in serotype distribution between hospitalized and outpatient CAAP cases. The results exemplify how the vaccine-probe approach can contribute to a better understanding of the pathogenesis of disease, but are by no means conclusive. Since February 2025, PCV20 has replaced PCV13 in the Israeli NIP. It remains to be seen whether the addition of the seven non-PCV13 serotypes will narrow the gap in PCV impact between outpatients versus hospitalized CAAP rates. It is important to emphasize that the current study was done on radiologically confirmed CAAP episodes. Although these are episodes that were typically considered likely to be bacterial, these are only a subset of all pneumonia cases, on the one hand, and pneumococcal pneumonia can occur with non-alveolar radiological findings or even without radiological findings, on the other hand. In addition, the study included children residing in a single geographical area (southern Israel) and the population consisted of 2 ethnic groups differing by socioeconomic and morbidity backgrounds. Thus, our findings cannot be automatically projected to all lower respiratory infections or non-alveolar pneumonia.

## Supplementary Material

ofaf710_Supplementary_Data

## Supplementary Data

Supplementary materials are available at *Open Forum Infectious Diseases* online. Consisting of data provided by the authors to benefit the reader, the posted materials are not copyedited and are the sole responsibility of the authors, so questions or comments should be addressed to the corresponding author.
