# Supplementary material for: Differential Impact of Pneumococcal Conjugate Vaccines on Hospitalized Versus Outpatient Community-Acquired Alveolar Pneumonia in Children Younger Than 5 Years Suggests Differences in Pathogenesis
Source: Open Forum Infect Dis. 2025 Nov 18;12(12):ofaf710. doi: 10.1093/ofid/ofaf710 (PMC12673847; doi:10.1093/ofid/ofaf710)
Supplement: ofaf710_Supplementary_Data [file ofaf710_supplementary_data.zip › Supplementary Table 2 .docx]

**Supplementary Table 2: Mean IRRs with 95% Confidence Intervals for every Vaccine Period *vs.* Expected episodes by Age Group and Care Setting)**

|  | **7/13 Transition period** | **Early PCV13 period** | **Late PCV 13 period** |
| --- | --- | --- | --- |
| **<12m** |  |  |  |
| Outpatients |  |  |  |
| Jewish | 0.57 (0.39; 0.81) | 0.39 (0.28; 0.53) | 0.42 (0.30; 0.58) |
| Bedouin | 0.68 (0.48; 0.94) | 0.28 (0.20; 0.38) | 0.41 (0.30; 0.55) |
| Overall | 0.63 (0.48; 0.81) | 0.32 (0.25; 0.42) | 0.41 (0.33; 0.52) |
| Hospitalized |  |  |  |
| Jewish | 1.06 (0.79; 1.39) | 0.71 (0.55; 0.91) | 0.83 (0.66; 1.08) |
| Bedouin | 1.06 (0.84; 1.31) | 0.59 (0.48; 0.72) | 0.53 (0.43; 0.65) |
| Overall | 1.05 (0.86; 1.26) | 0.62 (0.52; 0.73) | 0.60 (0.51; 0.72) |
| All episodes |  |  |  |
| Jewish | 0.87 (0.68; 1.09) | 0.58 (0.47; 0.71) | 0.68 (0.55; 0.84) |
| Bedouin | 0.98 (0.79; 1.20) | 0.53 (0.44; 0.63) | 0.50 (0.41; 0.60) |
| Overall | 0.94 (0.79; 1.12) | 0.54 (0.47; 0.63) | 0.56 (0.47; 0.65) |
| **12-23m** |  |  |  |
| Outpatients |  |  |  |
| Jewish | 0.54 (0.41; 0.69) | 0.33 (0.26; 0.41) | 0.35 (0.27; 0.44) |
| Bedouin | 0.57 (0.44; 0.73) | 0.31 (0.24; 0.40) | 0.29 (0.23; 0.37) |
| Overall | 0.56 (0.46; 0.66) | 0.32 (0.27; 0.38) | 0.32 (0.27; 0.38) |
| Hospitalized |  |  |  |
| Jewish | 1.19 (0.91; 1.50) | 0.60 (0.47; 0.75) | 0.61 (0.48; 0.76) |
| Bedouin | 0.94 (0.76; 1.14) | 0.48 (0.40; 0.58) | 0.46 (0.38; 0.56) |
| Overall | 1.02 (0.86; 1.20) | 0.53 (0.45; 0.61) | 0.51 (0.43; 0.60) |
| All episodes |  |  |  |
| Jewish | 0.78 (0.64; 0.94) | 0.43 (0.36; 0.51) | 0.45 (0.37; 0.53) |
| Bedouin | 0.80 (0.67; 0.94) | 0.42 (0.36; 0.49) | 0.40 (0.33; 0.47) |
| Overall | 0.79 (0.69; 0.90) | 0.43 (0.38; 0.48) | 0.42 (0.37; 0.47) |
| **24-59m** |  |  |  |
| Outpatients |  |  |  |
| Jewish | 0.79 (0.62; 0.98) | 0.37 (0.29; 0.46) | 0.26 (0.21; 0.33) |
| Bedouin | 0.87 (0.69; 1.11) | 0.43 (0.34; 0.55) | 0.36 (0.28; 0.45) |
| Overall | 0.83 (0.69; 0.98) | 0.40 (0.33; 0.46) | 0.30 (0.25; 0.36) |
| Hospitalized |  |  |  |
| Jewish | 1.20 (0.94; 1.51) | 0.58 (0.46; 0.73) | 0.42 (0.33; 0.53) |
| Bedouin | 1.26 (1.02; 1.54) | 0.65 (0.54; 0.79) | 0.42 (0.35; 0.51) |
| Overall | 1.23 (1.04; 1.45) | 0.63 (0.54; 0.73) | 0.42 (0.35; 0.50) |
| All episodes |  |  |  |
| Jewish | 0.86 (0.73; 1.00) | 0.48 (0.41; 0.54) | 0.44 (0.38; 0.51) |
| Bedouin | 1.00 (0.86; 1.16) | 0.52 (0.45; 0.60) | 0.45 (0.39; 0.51) |
| Overall | 0.94 (0.83; 1.05) | 0.50 (0.45; 0.56) | 0.45 (0.40; 0.49) |
