# Supplementary material for: Differential Impact of Pneumococcal Conjugate Vaccines on Hospitalized Versus Outpatient Community-Acquired Alveolar Pneumonia in Children Younger Than 5 Years Suggests Differences in Pathogenesis
Source: Open Forum Infect Dis. 2025 Nov 18;12(12):ofaf710. doi: 10.1093/ofid/ofaf710 (PMC12673847; doi:10.1093/ofid/ofaf710)
Supplement: ofaf710_Supplementary_Data [file ofaf710_supplementary_data.zip › Supplementary Table 1.docx]

**Supplementary Table 1: Mean Annual CAAP Incidence per 1,000 Children <60 Months Across Vaccine Periods (July 2004–June 2019, by Ethnicity, Age group and Care Setting)**

|  | **Outpatients** | | | **Hospitalized** | | | **Total** | | |
| --- | --- | --- | --- | --- | --- | --- | --- | --- | --- |
|  | **Jewish** | **Bedouin** | **Total** | **Jewish** | **Bedouin** | **Total** | **Jewish** | **Bedouin** | **Total** |
|  |  |  |  |  |  |  |  |  |  |
| **<12 months** |  |  |  |  |  |  |  |  |  |
|  |  |  |  |  |  |  |  |  |  |
| 2004-2005 | 4.5 | 6.3 | 5.4 | 6.1 | 29.4 | 17.4 | 10.6 | 35.8 | 22.8 |
| 2005-2006 | 3.6 | 4.2 | 3.9 | 8.4 | 27.1 | 17.4 | 12.0 | 31.3 | 21.3 |
| 2006-2007 | 4.1 | 8.6 | 6.2 | 7.7 | 34.5 | 20.5 | 11.8 | 43.0 | 26.7 |
| 2007-2008 | 6.5 | 5.0 | 5.8 | 10.0 | 26.5 | 17.9 | 16.5 | 31.5 | 23.7 |
| 2008-2009 | 3.5 | 6.2 | 4.8 | 8.7 | 33.2 | 20.5 | 12.2 | 39.4 | 25.3 |
| Mean 2004-2009 | 4.4 ± 1.2 | 6.1 ± 1.6 | 5.2 ± 0.9 | 8.2 ± 1.4 | 30.2 ± 3.6 | 18.8 ± 1.6 | 12.6 ± 2.3 | 36.2 ± 5.1 | 24 ± 2.1 |
| 2009-2010 | 2.2 | 4.8 | 3.5 | 11.1 | 30.2 | 20.4 | 13.3 | 35.0 | 23.9 |
| 2010-2011 | 2.8 | 3.4 | 3.1 | 8.2 | 31.8 | 19.7 | 11.0 | 35.2 | 22.8 |
| Mean 2009-2011 | 2.5 ± 0.4 | 4.1 ± 1 | 3.3 ± 0.3 | 9.6 ± 2.1 | 31 ± 1.1 | 20.1 ± 0.5 | 12.1 ± 1.7 | 35.1 ± 0.1 | 23.4 ± 0.7 |
| 2011-2012 | 2.5 | 0.9 | 1.7 | 5.7 | 18.6 | 12.0 | 8.1 | 19.4 | 13.7 |
| 2012-2013 | 1.0 | 1.1 | 1.1 | 5.2 | 18.1 | 11.5 | 6.2 | 19.2 | 12.5 |
| 2013-2014 | 1.6 | 2.0 | 1.8 | 6.1 | 14.3 | 10.1 | 7.7 | 16.3 | 11.9 |
| 2014-2015 | 1.9 | 2.6 | 2.2 | 7.3 | 18.3 | 12.8 | 9.2 | 20.9 | 15.0 |
| Mean 2011-2015 | 1.7 ± 0.6 | 1.7 ± 0.8 | 1.7 ± 0.5 | 6.1 ± 0.9 | 17.3 ± 2 | 11.6 ± 1.1 | 7.8 ± 1.2 | 19 ± 1.9 | 13.3 ± 1.4 |
| 2015-2016 | 3.0 | 3.3 | 3.1 | 6.0 | 18.8 | 12.3 | 8.9 | 22.0 | 15.4 |
| 2016-2017 | 1.5 | 2.3 | 1.9 | 8.7 | 22.2 | 15.4 | 10.2 | 24.5 | 17.3 |
| 2017-2018 | 1.1 | 2.2 | 1.6 | 6.2 | 14.8 | 10.6 | 7.2 | 17.0 | 12.2 |
| 2018-2019 | 2.0 | 2.4 | 2.2 | 8.2 | 14.8 | 11.7 | 10.2 | 17.2 | 13.9 |
| Mean 2015-2019 | 1.9 ± 0.8 | 2.5 ± 0.5 | 2.2 ± 0.6 | 7.3 ± 1.4 | 17.5 ± 3.6 | 12.5 ± 2.1 | 9.1 ± 1.4 | 20.1 ± 3.7 | 14.7 ± 2.2 |
|  |  |  |  |  |  |  |  |  |  |
| **12-23 months** |  |  |  |  |  |  |  |  |  |
|  |  |  |  |  |  |  |  |  |  |
| 2004-2005 | 10.6 | 7.7 | 9.2 | 5.3 | 10.5 | 7.9 | 15.9 | 18.2 | 17.0 |
| 2005-2006 | 10.4 | 7.2 | 8.9 | 7.7 | 13.4 | 10.5 | 18.1 | 20.6 | 19.3 |
| 2006-2007 | 10.0 | 9.2 | 9.6 | 6.3 | 13.4 | 9.7 | 16.3 | 22.5 | 19.3 |
| 2007-2008 | 12.8 | 8.4 | 10.7 | 8.0 | 13.4 | 10.6 | 20.8 | 21.8 | 21.3 |
| 2008-2009 | 7.7 | 6.3 | 7.0 | 6.4 | 14.9 | 10.5 | 14.1 | 21.1 | 17.5 |
| Mean 2004-2009 | 10.3 ± 1.8 | 7.7 ± 1.1 | 9.1 ± 1.4 | 6.7 ± 1.1 | 13.1 ± 1.6 | 9.8 ± 1.1 | 17 ± 2.5 | 20.8 ± 1.6 | 18.9 ± 1.7 |
| 2009-2010 | 5.5 | 5.1 | 5.3 | 8.1 | 14.3 | 11.1 | 13.6 | 19.4 | 16.4 |
| 2010-2011 | 5.8 | 3.7 | 4.8 | 8.5 | 9.9 | 9.2 | 14.3 | 13.6 | 13.9 |
| Mean 2009-2011 | 5.7 ± 0.2 | 4.4 ± 1 | 5.1 ± 0.4 | 8.3 ± 0.3 | 12.1 ± 3.1 | 10.1 ± 1.3 | 14 ± 0.5 | 16.5 ± 4.1 | 15.2 ± 1.7 |
| 2011-2012 | 4.5 | 3.3 | 3.9 | 3.9 | 6.6 | 5.2 | 8.4 | 9.9 | 9.1 |
| 2012-2013 | 3.7 | 2.5 | 3.1 | 4.0 | 7.3 | 5.6 | 7.7 | 9.7 | 8.7 |
| 2013-2014 | 1.8 | 2.7 | 2.2 | 5.5 | 6.8 | 6.1 | 7.4 | 9.5 | 8.4 |
| 2014-2015 | 3.4 | 1.3 | 2.4 | 3.3 | 5.1 | 4.2 | 6.7 | 6.4 | 6.5 |
| Mean 2011-2015 | 3.3 ± 1.1 | 2.4 ± 0.8 | 2.9 ± 0.8 | 4.2 ± 1 | 6.4 ± 0.9 | 5.3 ± 0.8 | 7.5 ± 0.7 | 8.8 ± 1.7 | 8.2 ± 1.1 |
| 2015-2016 | 3.6 | 2.7 | 3.2 | 4.1 | 8.1 | 6.1 | 7.7 | 10.8 | 9.3 |
| 2016-2017 | 3.5 | 3.0 | 3.3 | 4.0 | 5.2 | 4.6 | 7.6 | 8.2 | 7.9 |
| 2017-2018 | 3.6 | 2.0 | 2.8 | 4.5 | 6.3 | 5.4 | 8.1 | 8.3 | 8.2 |
| 2018-2019 | 3.5 | 1.5 | 2.5 | 4.4 | 5.3 | 4.9 | 7.9 | 6.8 | 7.4 |
| Mean 2015-2019 | 3.6 ± 0.1 | 2.3 ± 0.7 | 2.9 ± 0.4 | 4.3 ± 0.2 | 6.2 ± 1.4 | 5.2 ± 0.6 | 7.8 ± 0.2 | 8.5 ± 1.7 | 8.2 ± 0.8 |
|  |  |  |  |  |  |  |  |  |  |
| **24-59 months** |  |  |  |  |  |  |  |  |  |
|  |  |  |  |  |  |  |  |  |  |
| 2004-2005 | 4.2 | 3.3 | 3.7 | 1.9 | 4.5 | 3.2 | 6.1 | 7.8 | 6.9 |
| 2005-2006 | 4.6 | 3.6 | 4.1 | 2.8 | 6.3 | 4.5 | 7.4 | 9.9 | 8.6 |
| 2006-2007 | 4.1 | 3.1 | 3.6 | 3.0 | 4.4 | 3.7 | 7.1 | 7.5 | 7.3 |
| 2007-2008 | 5.4 | 3.3 | 4.4 | 3.7 | 4.5 | 4.1 | 9.1 | 7.9 | 8.5 |
| 2008-2009 | 3.8 | 3.4 | 3.6 | 2.8 | 4.9 | 3.8 | 6.6 | 8.3 | 7.4 |
| Mean 2004-2009 | 4.4 ± 0.6 | 3.3 ± 0.2 | 3.9 ± 0.4 | 2.9 ± 0.6 | 5 ± 0.8 | 3.9 ± 0.5 | 7.3 ± 1.1 | 8.3 ± 1 | 7.8 ± 0.8 |
| 2009-2010 | 3.8 | 3.4 | 3.6 | 4.0 | 6.9 | 5.4 | 7.8 | 10.2 | 8.9 |
| 2010-2011 | 3.1 | 2.5 | 2.8 | 3.0 | 5.3 | 4.1 | 6.1 | 7.8 | 6.9 |
| Mean 2009-2011 | 3.4 ± 0.5 | 2.9 ± 0.6 | 3.2 ± 0.6 | 3.5 ± 0.7 | 6.1 ± 1.1 | 4.7 ± 0.9 | 6.9 ± 1.2 | 9 ± 1.8 | 7.9 ± 1.5 |
| 2011-2012 | 2.2 | 1.8 | 2.0 | 2.7 | 3.8 | 3.2 | 4.9 | 5.6 | 5.2 |
| 2012-2013 | 1.6 | 1.5 | 1.5 | 1.4 | 3.7 | 2.5 | 3.0 | 5.2 | 4.1 |
| 2013-2014 | 1.6 | 1.4 | 1.5 | 1.4 | 3.0 | 2.2 | 3.0 | 4.4 | 3.7 |
| 2014-2015 | 1.1 | 1.1 | 1.1 | 1.0 | 2.5 | 1.7 | 2.2 | 3.6 | 2.9 |
| Mean 2011-2015 | 1.6 ± 0.4 | 1.4 ± 0.3 | 1.5 ± 0.4 | 1.6 ± 0.7 | 3.2 ± 0.6 | 2.4 ± 0.6 | 3.3 ± 1.2 | 4.7 ± 0.9 | 4 ± 1 |
| 2015-2016 | 1.5 | 1.3 | 1.4 | 1.0 | 2.9 | 1.9 | 2.5 | 4.1 | 3.3 |
| 2016-2017 | 0.9 | 1.5 | 1.2 | 1.4 | 2.0 | 1.7 | 2.3 | 3.4 | 2.8 |
| 2017-2018 | 0.7 | 1.1 | 0.9 | 0.7 | 1.8 | 1.3 | 1.4 | 2.9 | 2.2 |
| 2018-2019 | 1.5 | 1.0 | 1.2 | 1.7 | 2.0 | 1.8 | 3.2 | 3.0 | 3.1 |
| Mean 2015-2019 | 1.1 ± 0.4 | 1.2 ± 0.2 | 1.2 ± 0.2 | 1.2 ± 0.4 | 2.1 ± 0.5 | 1.7 ± 0.3 | 2.4 ± 0.7 | 3.4 ± 0.6 | 2.8 ± 0.5 |
