# Supplementary figures and images for: Differential Impact of Pneumococcal Conjugate Vaccines on Hospitalized Versus Outpatient Community-Acquired Alveolar Pneumonia in Children Younger Than 5 Years Suggests Differences in Pathogenesis
Source: Open Forum Infect Dis. 2025 Nov 18;12(12):ofaf710. doi: 10.1093/ofid/ofaf710 (PMC12673847; doi:10.1093/ofid/ofaf710)

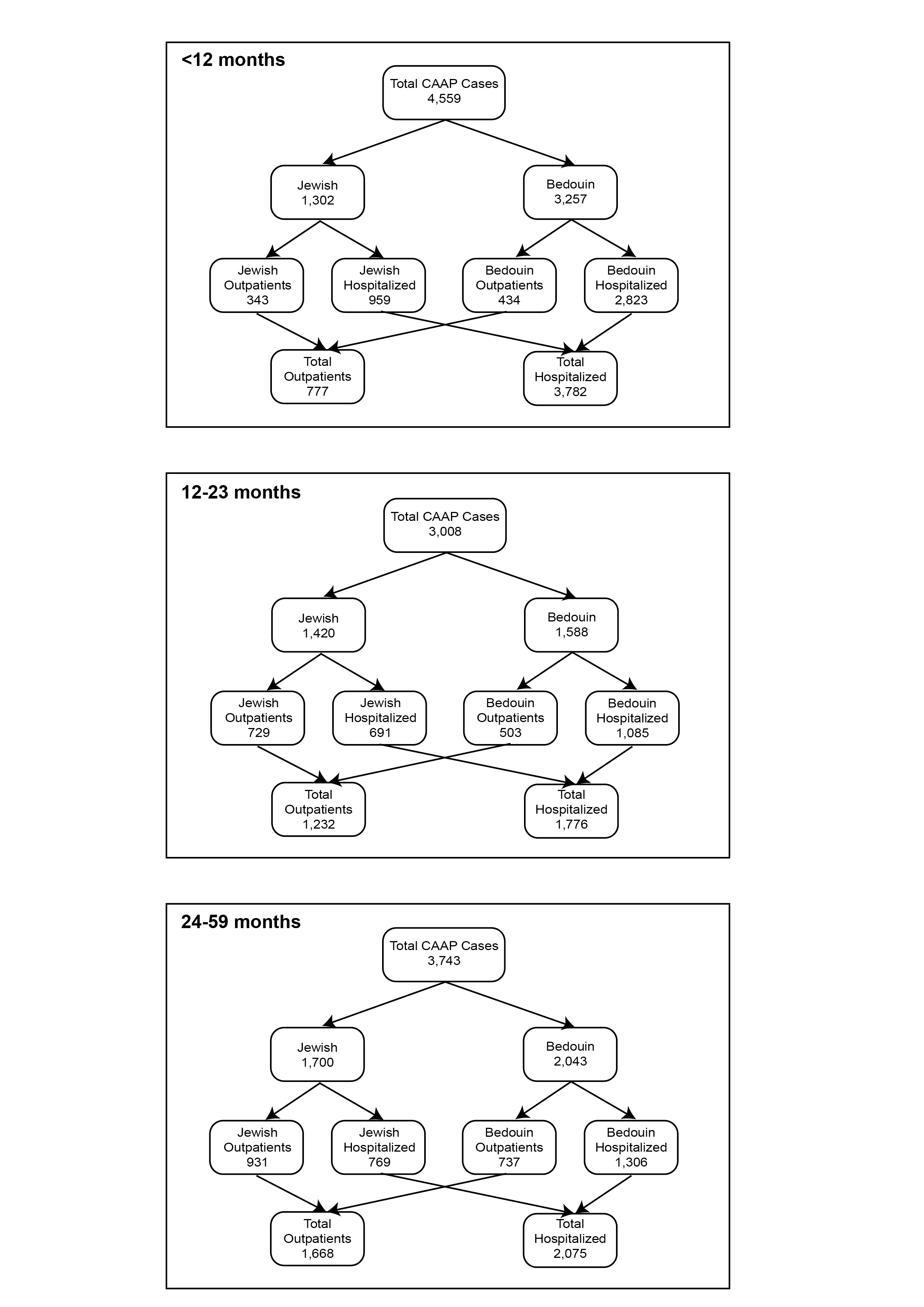

Supplement: ofaf710_Supplementary_Data [file ofaf710_supplementary_data.zip › Supplementary Figure 1.png]

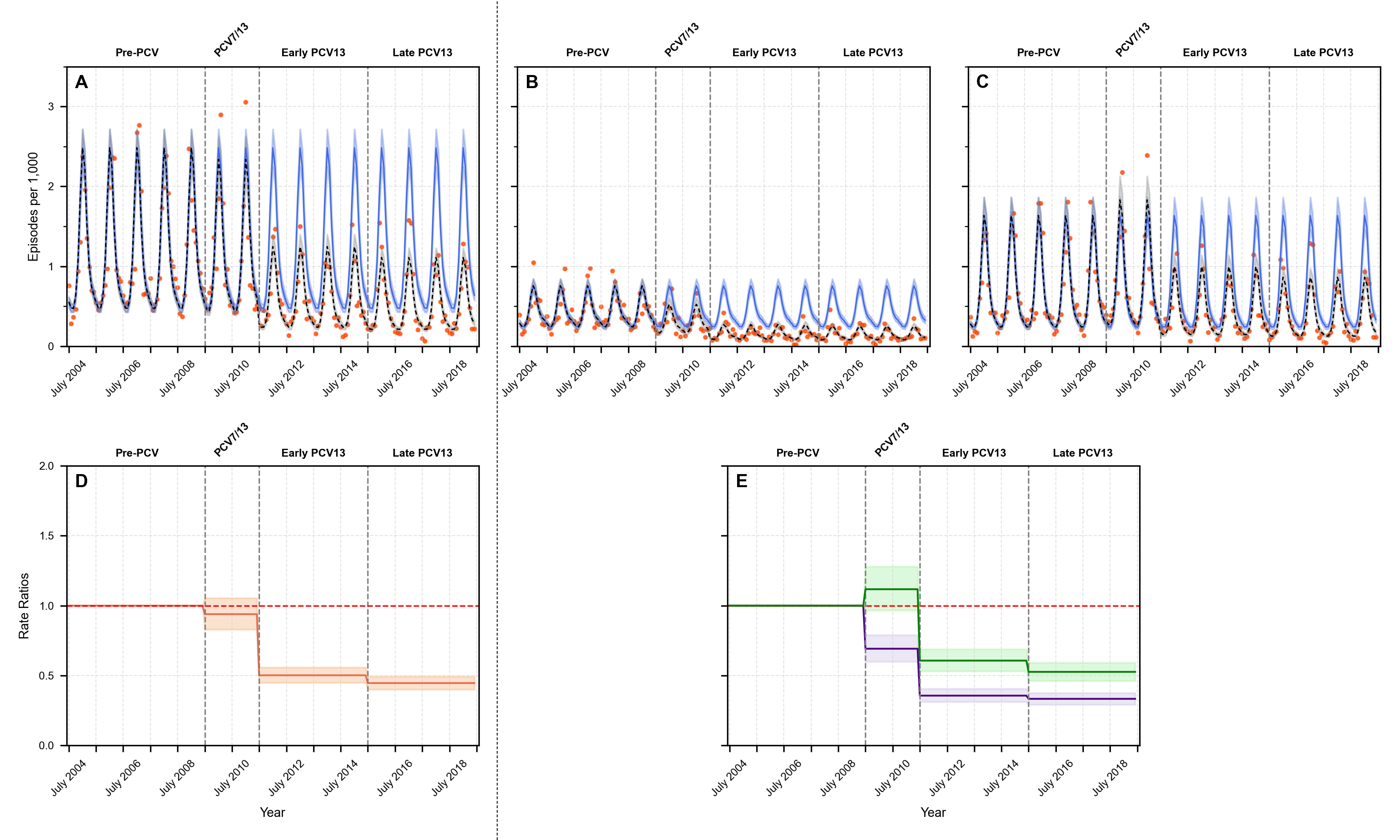

Supplement: ofaf710_Supplementary_Data [file ofaf710_supplementary_data.zip › Supplementary Figure 2.png]

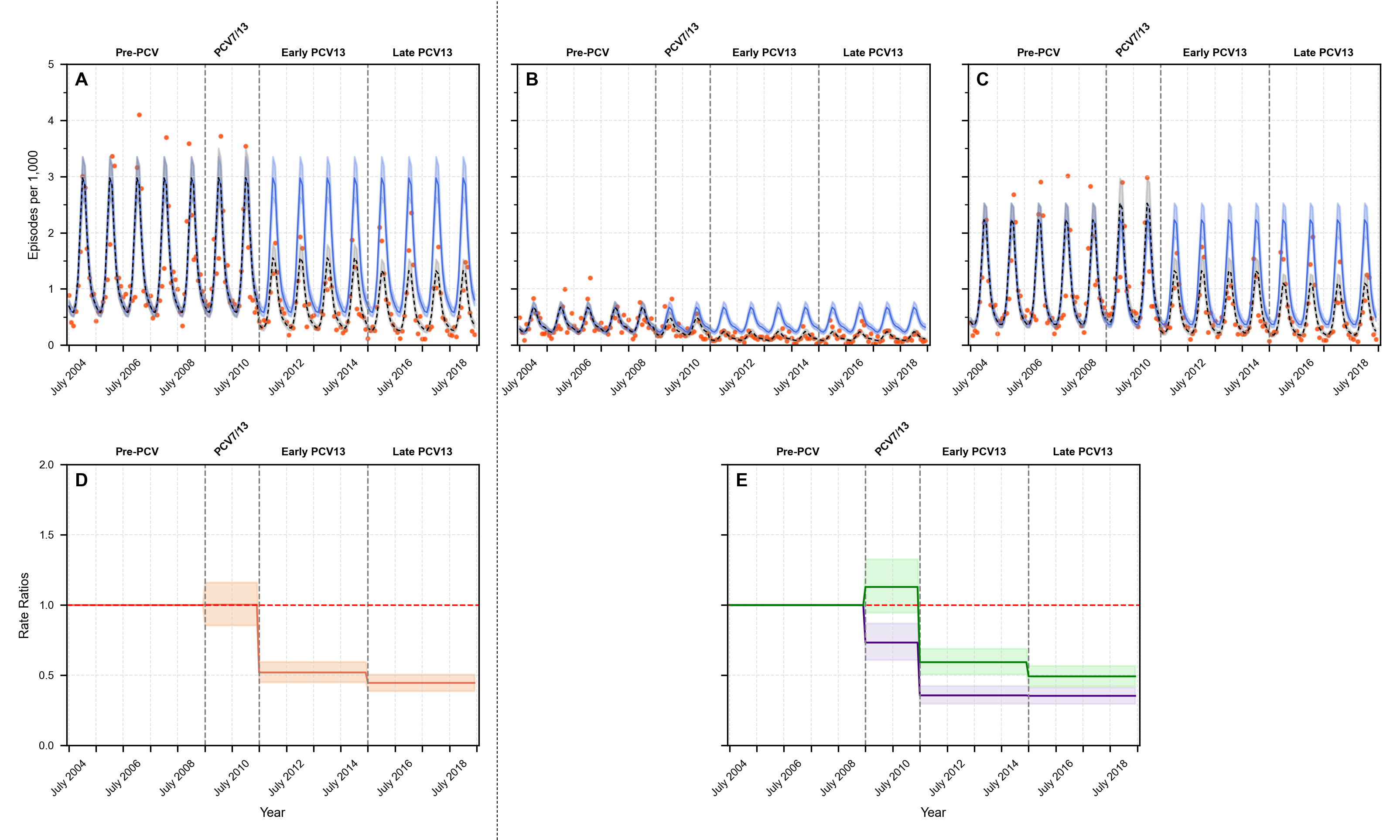

Supplement: ofaf710_Supplementary_Data [file ofaf710_supplementary_data.zip › Supplementary Figure 3.png]

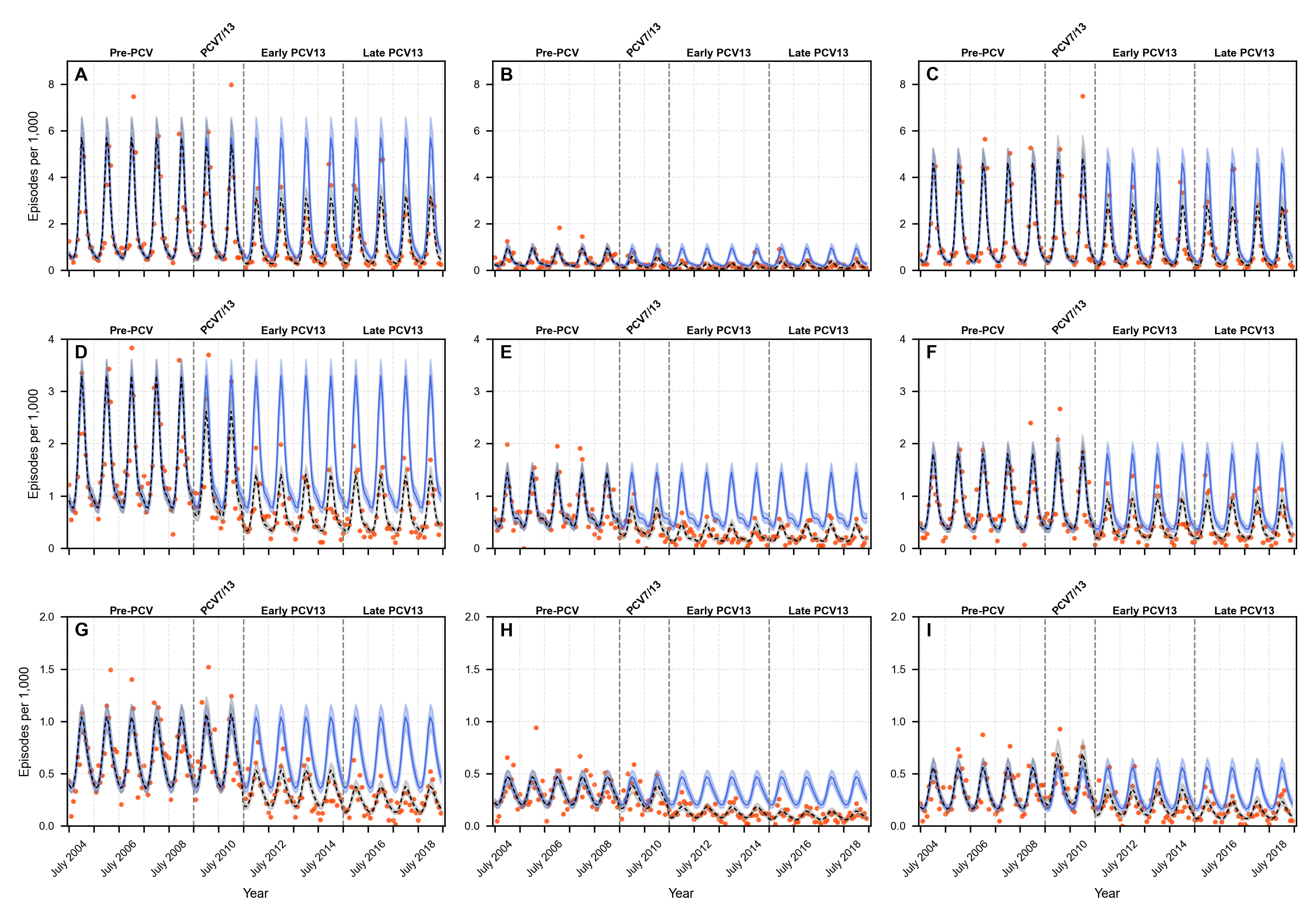

Supplement: ofaf710_Supplementary_Data [file ofaf710_supplementary_data.zip › Supplementary Figure 4.png]

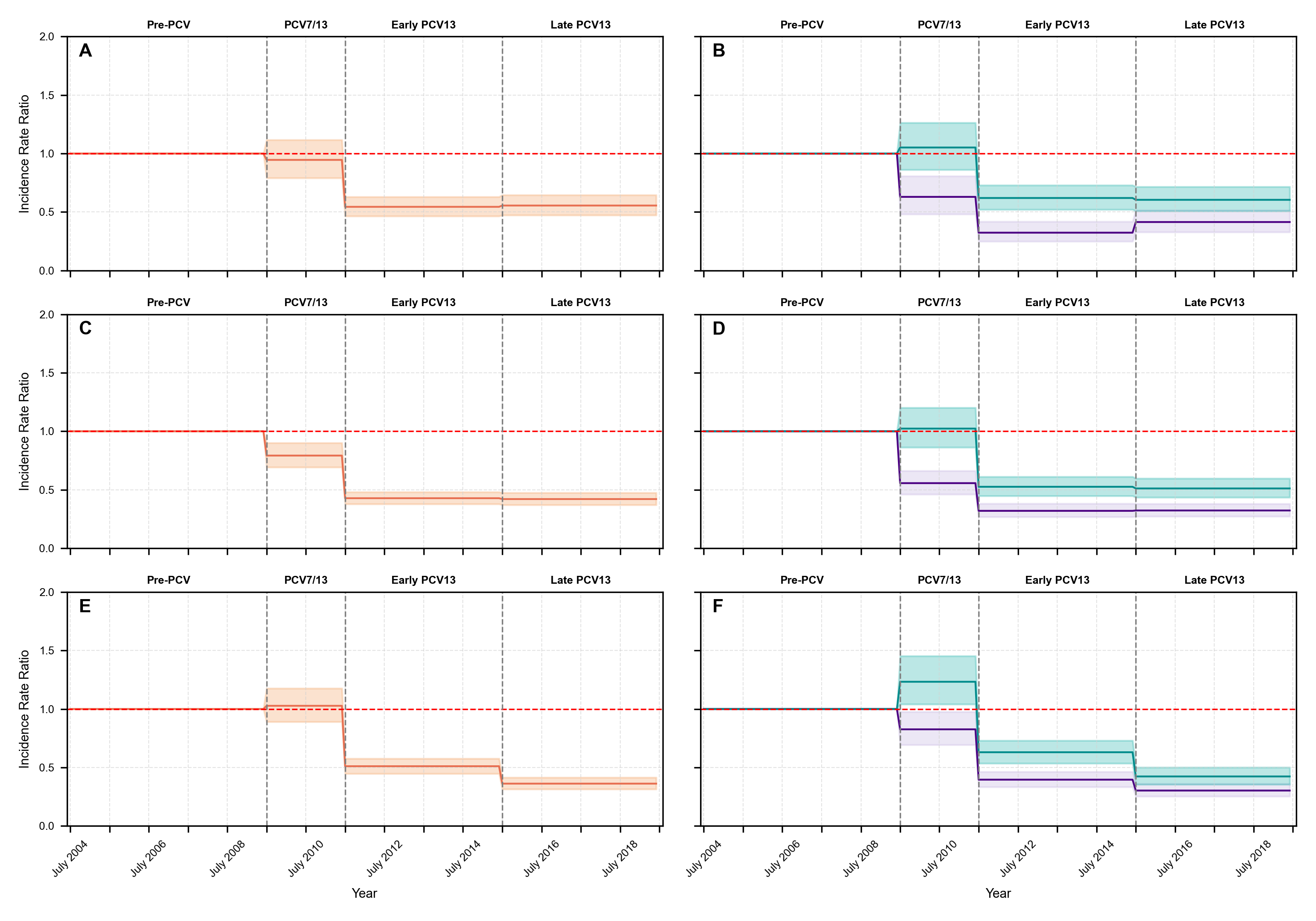

Supplement: ofaf710_Supplementary_Data [file ofaf710_supplementary_data.zip › Supplementary Figure 5.png]

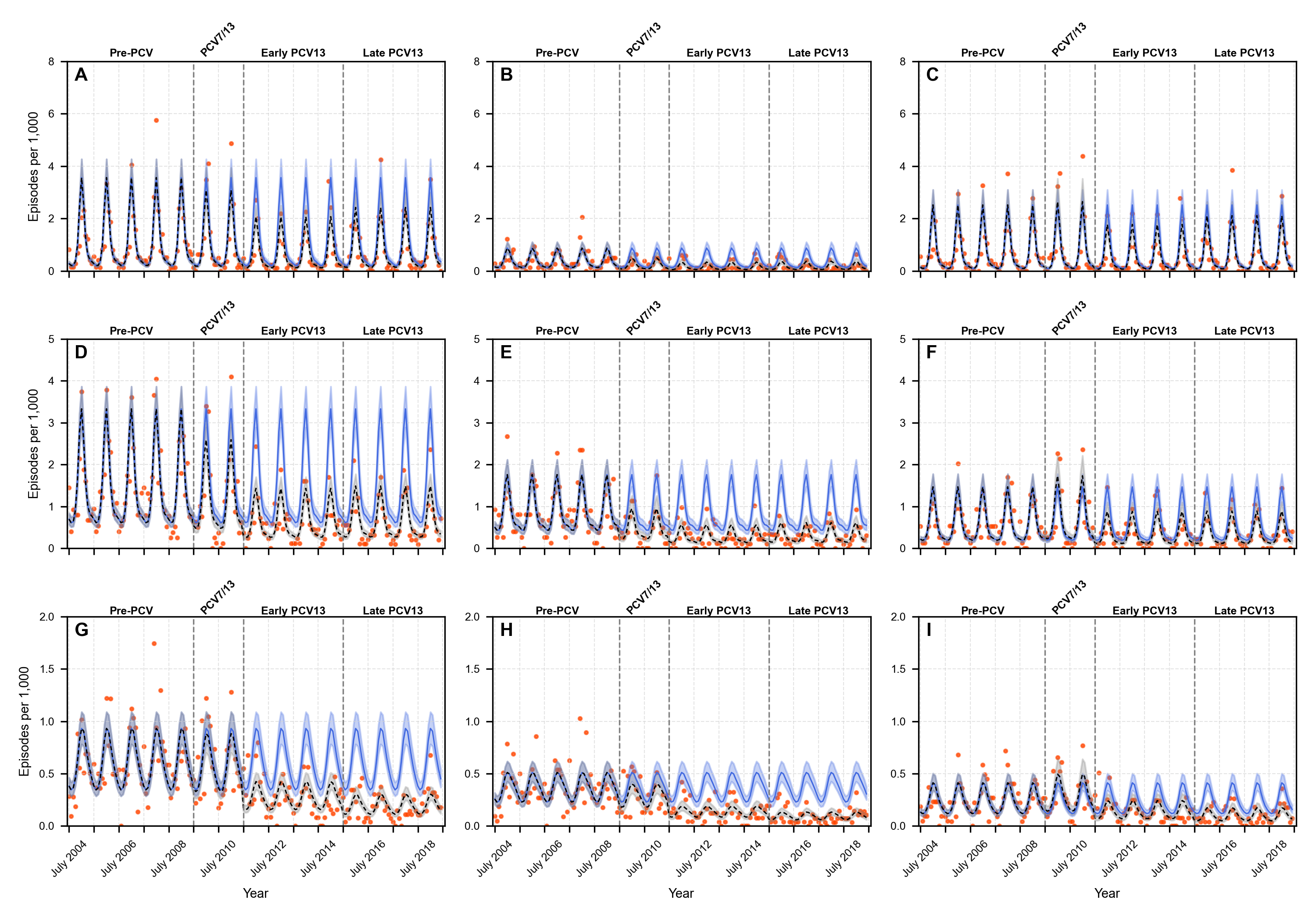

Supplement: ofaf710_Supplementary_Data [file ofaf710_supplementary_data.zip › Supplementary Figure 6.png]

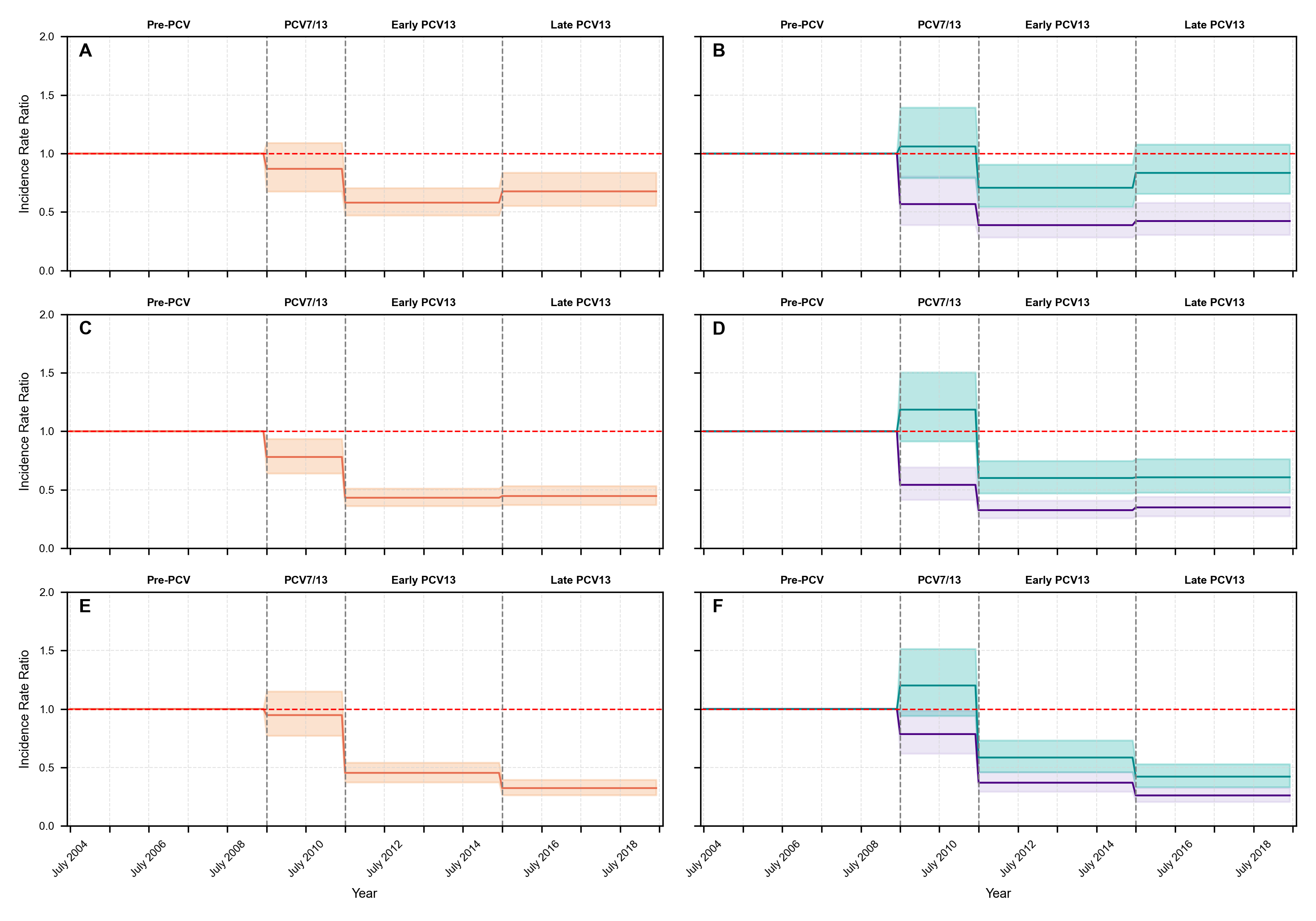

Supplement: ofaf710_Supplementary_Data [file ofaf710_supplementary_data.zip › Supplementary Figure 7.png]

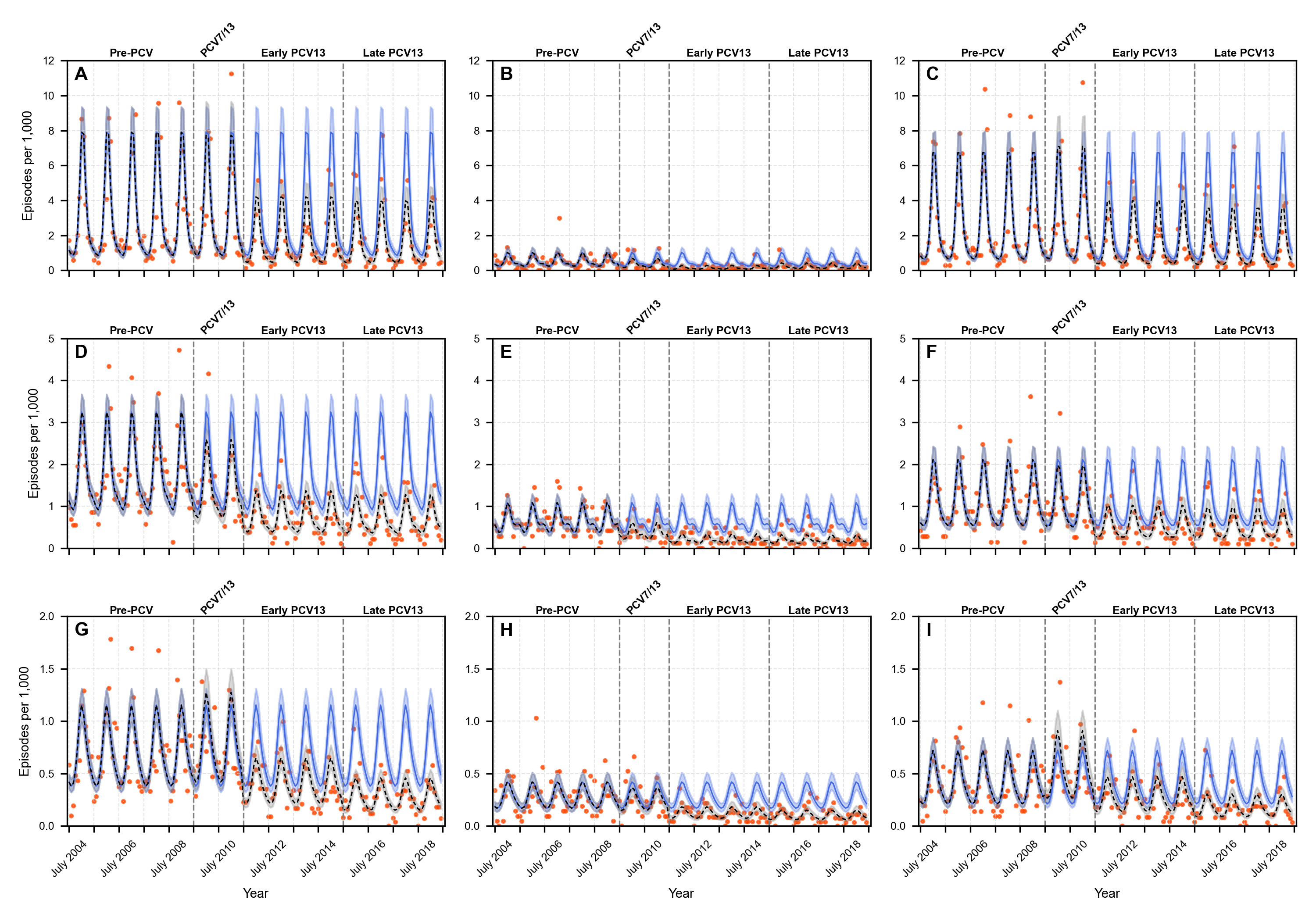

Supplement: ofaf710_Supplementary_Data [file ofaf710_supplementary_data.zip › Supplementary Figure 8.png]

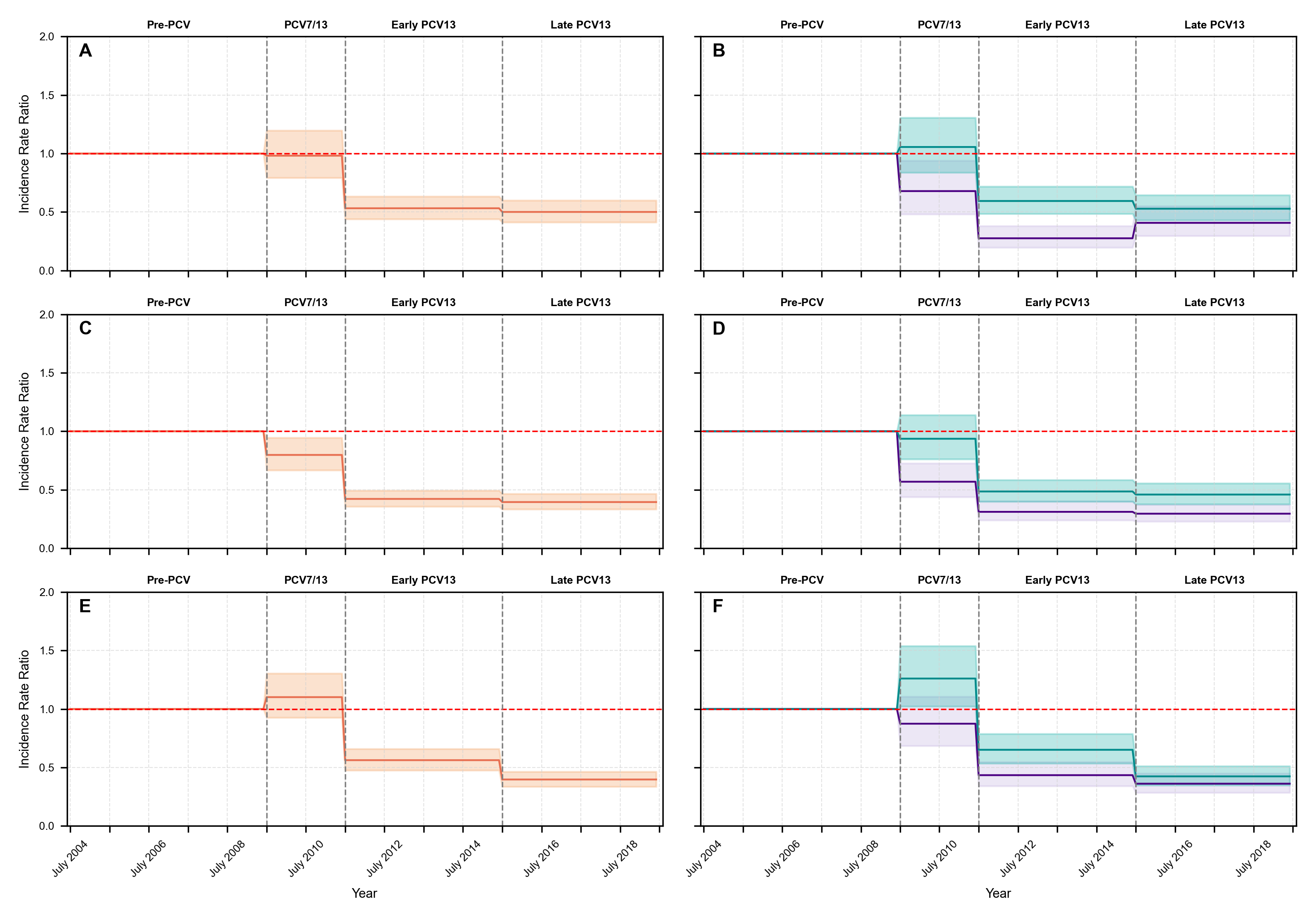

Supplement: ofaf710_Supplementary_Data [file ofaf710_supplementary_data.zip › Supplementary Figure 9.png]
